# Contralateral facial nerve palsy following mandibular second molar removal: is there co-relation or just coincidence?

**DOI:** 10.11604/pamj.2014.18.173.3750

**Published:** 2014-06-22

**Authors:** Mohammed Zalagh, Ali Boukhari, Hicham Attifi, Mounir Hmidi, Abdelhamid Messary

**Affiliations:** 1 Service d'Otorhinolaryngologie, et de Chirurgie Cervico-faciale de l'Hôpital Militaire Moulay Ismaïl, Meknès, Maroc

**Keywords:** Facial nerve palsy, anaesthetic injection, molar removal

## Abstract

Peripheral facial nerve palsy (FNP) is the most common cranial nerves neuropathy. It is very rare during dental treatment. Classically, it begins immediately after the injection of local anaesthetic into the region of inferior dental foramen and it's homolateral to the injection. Recovery takes a few hours, normally as long the anaesthetic lasts. The authors present a 44-year-old patient who presented a contralateral delayed-onset facial paralysis arising from dental procedure and discuss the plausible pathogenesis mechanism of happen and a possible relationship between dental procedure and contralateral FNP.

## Introduction

Peripheral facial palsy (PFP) is the most common cranial nerves neuropathy. It may be central or peripheral, complete or incomplete. On quarter of PFP are secondary, with possible causes including, trauma, surgery, local infections, tumor, and stroke. However, the majority of cases (75% [1, 2]) remain without and identified aetiology and will eventually be idiopathic PFP, or Bell's palsy. PFP during dental treatment is very rare. It begins immediately after the injection of local anaesthetic into the region of inferior dental foramen and it's homolateral to the injection. Recovery takes a few hours, normally as long the anaesthetic lasts. Literature reports usually describe a sudden onset of homolateral PFP after dental procedure. In this paper, the authors report a rare case of contralateral delayed-onset PFP following dental procedure which didn't recovery over 9 months of follow up and discus the likely mechanism pathogenic of this rare case and the cause effect relationship between dental procedure and contralateral PFP.

## Patient and observation

A 44 year-old diabetic mellitus man was referred to the oral surgeon to have his left lower second molar extraction which was decayed. The injection of the anesthetic solution was uneventful. However, the molar extraction was prolonged and marked by hemorrhage and the maneuver was so long. There was no sign, of facial paralysis at the end of the surgical procedure. His past medical history included previous difficult superior left wisdom tooth extraction. But, there was no past facial paralysis following a dental procedure.

Eight hours later, the patient noticed the sudden onset of contralateral facial weakness associated to hyperacusis, loss of taste of the anterior two-thirds of his tongue, excess lacrimination affecting the right eye that couldn't close spontaneously. The oral surgeon prescribed a Vitamin B1 B6 B12 complex and analgesics, and referred the patient to our department.

On his admission, physical examination disclosed a well-appearing, alert and afebrile man, who presented a complete right-side peripheral facial palsy, assessed as Grade III according to the House-brackmann grading system. The remainder of the full neurological examination was normal. There was no deafness and bilateral otoscopy was normal. Also, no herpetic vesicles were found in the retro-auricular area. General examination was unremarkable. MRI didn't find any intra-cranial lesion. Blood tests were all within normal ranges. The electromyographic studies gave the diagnosis of complete peripheral palsy with absence of denervation.

The treatment consisted of regimen of Prednisone (2mg/kg/day) and artificial tears for 10 days with the dose being gradually reduced. Despite of early treatment with Prednisone, his palsy persisted for 9 months without substantial clinical improvement. The patient still complaining of residual facial muscles weakness, but the sensation and tearing disappeared.

## Discussion

The previous clinical observation suggests that our patient presented a complete PFP after left lower second molar extraction. Classically, PFP related to dental intervention is homolateral to injection, has sudden-onset, and can be caused by three mechanisms: direct trauma to the nerve from needle, intraneural hematoma formation or compression by hematoma or /and oedema in the region of the parotid gland, and local anaesthetic toxicity injected in the proximity of parotid [3, 4]. Such local mechanisms cannot explain the involvement of the delayed and contralateral PFP in our case. The presence of hypersensitivity to sound, reduction in taste and numbness around the ear on the side affected in our patient suggests involvement of the nerve proximal to its exit from the stylomastoid foramen with the sudden-onset evocate idiopathic palsy. Furthermore, nor other evident structural lesion in the ear or parotid gland (cholesteatoma, acute otitis media, Lyme disease, parotid tumor, sarcoidosis) neither influenza vaccines was retained in the case to explain this paralysis. These lesions usually have additional features that distinguish them from idiopathic palsy. However, the comprehensive literature review of different way to define Bell's palsy has shown that standard diagnostic criteria are not available [5] and the pathogenesis of Bell's palsy still unknown. Ischemia, autoimmune demyelinisation or viral reactivations are suggested as possible mechanisms [5]. In fact, the cause has been shown to be (in cases formerly labelled as “idiopathic”) viral [6] with associated ischemia and compression of the facial nerve in the narrow confines of its course through the temporal bone [6]. It has been suggested that the reactivation of herpes *simplex virus* (HSV) genome from geniculate ganglia is the likely pathogenesis of Bell's palsy [7].

Effectively, latent HSV-1 has been detected by polymerase chain reaction (PCR) or by histology in 70% to 90% of unselected geniculate ganglia collected to autopsy [8]. Regarding the biological and histological studies [9], the HSV-1 reactivation might induce local inflammation following immunologic process (anti-viral and anti-myelin antibodies) that favours phenomenal compression and ischemia resulting in paralysis. Animal model findings suggest that a combination of stimuli, local irritation skin, and general immunosuppression is essential (required as factors to induce) to successfully inducing facial nerve paralysis in nice with latent HSV-1 infection [7].

Retrospectively, we learned that our patient was so tired and stressed the day of surgery because he didn't sleep well day before and was scared to get dental extraction having in memory the last bad history of the previous wisdom tooth extraction. Immune compromise following health state patient (diabetes, stress, tiredness), hemorrhagic and longer maneuver extraction, seems to be a plausible explanation for virus reactivation resulting in PFP described in the described case.

## Conclusion

If dental procedure is retained as the plausible aetiology or predisposing factor of this facial paralysis, this case should emphasize the crucial requirement for a check list of the immunodeficient competent diseases, health state patient and premedication in some psychological profiles, to avoid facial paralysis following oral procedure.
